# Hemogram parameters can predict in-hospital mortality of patients with Myasthenic crisis

**DOI:** 10.1186/s12883-021-02412-4

**Published:** 2021-10-06

**Authors:** Che-Wei Hsu, Nai-Ching Chen, Wei-Chin Huang, Hui-Chen Lin, Wan-Chen Tsai, Chih-Cheng Huang, Ben-Chung Cheng, Nai-Wen Tsai

**Affiliations:** 1 Department of Neurology, Kaohsiung Chang Gung Memorial Hospital, Chang Gung University College of Medicine, No. 123, Ta Pei Road, Niao Sung District, Kaohsiung, 833 Taiwan; 2 Department of Nephrology, Kaohsiung Chang Gung Memorial Hospital, Chang Gung University College of Medicine, Kaohsiung, Taiwan

**Keywords:** Myasthenic crisis, Anemia, In-hospital mortality

## Abstract

**Background:**

Myasthenia gravis (MG) is an autoimmune disease involving the neuromuscular junction. Myasthenic crisis (MC), which is characterized by respiratory failure and the requirement of mechanical ventilation in patients with MG, is still a medical emergency despite the decrease in mortality with the advances in acute management. Hemogram is a cost-effective test for evaluating hematological complications and systemic inflammation, and hemogram data have been used to predict various clinical outcomes of several diseases. The relationship between hemogram and MG has been discussed, but the role of hemogram data in predicting the prognosis of MC patients has not been established.

**Methods:**

To identify whether hemogram data can predict in-hospital mortality in patients with MC, we retrospectively investigated 188 myasthenic crisis events from the Chang Gung Research Database between April 2001 and March 2019. Demographic and clinical characteristics were collected, as well as hemogram data before intubation and extubation. The endpoints were mortality during mechanical ventilation and mortality after extubation.

**Results:**

The overall in-hospital mortality rate was 22%. Multivariate logistic regression analysis for predicting mortality during mechanical ventilation showed that old age at MC onset (OR = 1.039, *p* = 0.022), moderate-to-severe anemia (OR = 5.851, *p* = 0.001), and extreme leukocytosis (OR = 5.659, *p* = 0.022) before intubation were strong predictors of mortality, while acute management with plasma exchange or double-filtration plasmapheresis (PE/DFPP) significantly decreased mortality (OR = 0.236, *p* = 0.012). For predicting mortality after extubation, moderate-to-severe anemia before extubation (OR = 8.452, *p* = 0.017) and non-treated with disease-modifying therapy before MC (OR = 5.459, *p* = 0.031) were crucial predictive factors.

**Conclusion:**

This study demonstrated that both old age at MC onset and moderate-to-severe anemia are important predictors of in-hospital mortality in patients with MC, and extreme leukocytosis is another crucial predictor of mortality during mechanical ventilation. The suggested mechanism is that anemia-induced hypoxia may enhance the release of proinflammatory cytokines, exacerbate systemic inflammation, and lead to multiple organ dysfunction syndrome and, finally, mortality.

## Introduction

Myasthenia gravis (MG) is an autoimmune disease caused by autoantibodies against nicotinic acetylcholine receptors or related molecules on the postsynaptic muscle membrane of the neuromuscular junction [1]. The major clinical manifestation of MG is weakness with fluctuating severity and fatigability involving different muscle groups, including the respiratory and bulbar muscles [2]. Myasthenic crisis (MC) is a medical emergency characterized by respiratory failure and the requirement of mechanical ventilation because of acute deterioration of respiratory dysfunction and bulbar muscle weakness in MG patients [3]. MG patients who received surgical procedures but experienced delayed extubation of >24 h at post-operative period were also considered to be in MC [4–6]. Approximately 15–20% of MG patients have suffered MC at least once in their lives [7]. The mortality rate of MC was 42% in the early 1960s [8], but it dramatically decreased to 4% in the 2000s [9] with advances in mechanical ventilation and acute immunomodulatory therapies [10], such as intravenous immunoglobulin, plasma exchange, and plasmapheresis [11, 12].

Hemogram is cost-effective and easily available for evaluating hematological complications (e.g., anemia, leukocytosis, and thrombocytopenia) in critically ill patients [13, 14]. Hematological complications have been identified as prognostic factors of mortality for various critical illnesses, including acute myocardial infarction [15, 16], infectious endocarditis [17], acute exacerbation of chronic obstructive pulmonary disease [18, 19], acute kidney injury [20], acute ischemic stroke [21], and community-acquired pneumonia [22]. Except for the number of different blood cells, the hemogram also provides several hemogram-derived indices, such as neutrophil-to-lymphocyte ratio (NLR), lymphocyte-to-monocyte ratio, and platelet-to-lymphocyte ratio. These indices are considered markers of systemic inflammation and are objective predictors of detrimental outcomes of septic shock [23], cancer treatment [24], and carotid endarterectomy [25].

Although hematological complications and the use of hemogram-derived indices have been reported in several critical illnesses, the relationship between hemogram findings and MG has been discussed in only a few articles [26, 27]. In this study, we focused on in-hospital mortality of MG patients with respiratory failure and hypothesized that hemograms can predict mortality at different stages of MC.

## Methods

### Data source

In this retrospective study, we established a cohort of patients with myasthenic crisis using patient information from the Chang Gung Research Database (CGRD) [28], which is an electronic medical record database of Chang Gung Memorial Hospital (CGMH). In Taiwan, CGMH is the largest private hospital system, which contains three medical centers (Taipei, Linku, and Kaohsiung branches) and four regional hospitals (Keelung, Taoyuan, Yunlin, and Chiayi branches). The CGRD data include demographic information, diagnostic codes of the International Classification of Diseases (ICD), medical records of drugs and special procedures, and the results of laboratory tests, image studies, and special examinations of inpatients and outpatients.

### Myasthenic crisis events

**Fig. 1:**
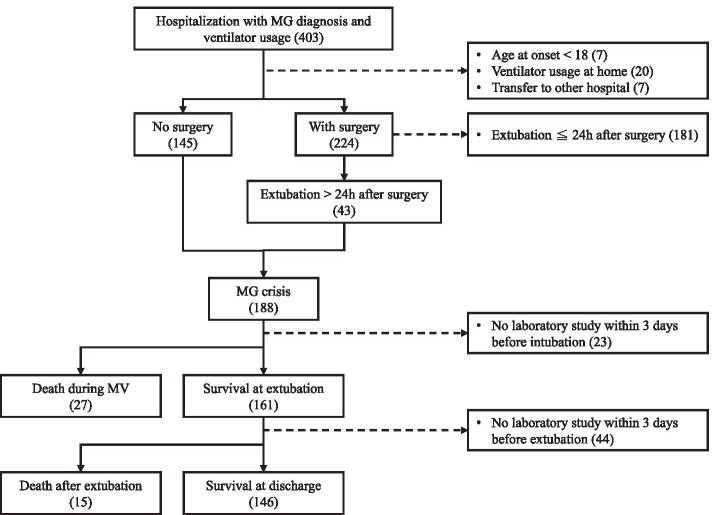
Flow chart for the enrollment of myasthenic crisis cases

Figure 1 demonstrates the search strategy for myasthenic crisis (MC) events in the CGRD. Initially, we searched for MG patients who had been admitted to one of the hospitals in the CGMH system between April 2001 and March 2019, using the ICD codes of MG with or without acute exacerbation (Ninth Clinical Modification [ICD-9-CM] 358.0x or 10^th^ Revision [ICD-10] G70.0x). Based on the records of respiratory therapists, evidence of ventilator usage during hospitalization was found in 403 events.

During the secondary step, we identified MC events in adult patients based on the above preliminary results. A MC event was defined as an acute deterioration of respiratory function in MG patients requiring mechanical ventilation. In this study, a MC event was also defined as prolonged ventilator support for more than 24 h after surgery in MG patients who underwent intubation for surgical purposes [4, 5]. Based on these definitions, we excluded 181 ventilator usage events for perioperative care within 24 h after surgery. Thirty-four other events were also excluded because of age at onset of < 18 years, tracheostomy with ventilator usage at home before admission, or family request for transfer to another hospital for critical care. A total of 188 MC events were enrolled in this study.

### Medical information of MC events

From the CGRD data of the 188 MC events, we obtained the gender and age at MC onset from the demographic information, underlying diseases and acute comorbidities from the ICD codes at discharge, and medications for MG treatment before admission and acute MC management from the medical records for the drugs and special procedures. Hemogram within 3 days before intubation was checked for 165 MC events, and we collected the latest hemogram data before intubation. Among the 161 MC events patients survived at extubation, the hemogram data within 3 days before extubation were available for 117 events, and we also collected the latest hemogram data before extubation.

### Definition of abnormalities in hemogram parameters

From the hemogram data, we extracted the original values of different parameters, including hemoglobin (Hb), white blood count (WBC), and platelet count (PC). Abnormalities in the hemogram parameters were further categorized according to the definition of the World Health Organization [29] and other previous studies [30, 31]. For the analysis in this study, we defined moderate anemia as Hb of 8-10.9 g/dL, severe anemia as Hb of < 8 g/dL, leukocytosis as WBC of > 10 x 10^9^ cells/L, extreme leukocytosis as WBC of > 25 x 10^9^ cells/L, and thrombocytopenia as platelet count of < 150 x 10^9^ cells/L. The differential count of WBC was also used to calculate theneutrophil-lymphocyte ratio (NLR).

### Statistical analysis

The results of numerical and categorical data are presented as mean ± SD and number (n), respectively. Because survival and mortality are mutual endpoints in every MC event, we viewed 188 MC events as 188 independent cases for analytic purposes. First, descriptive statistics were performed to analyze the demographic factors and clinical characteristics of the 188 cases. Second, 165 patients who had a hemogram within 3 days before intubation were separated into two groups (mortality during mechanical ventilation [M_dMV] vs. survival during mechanical ventilation [S_dMV]), while 117 patients who had hemograms within 3 days before extubation were further divided into paired subgroups (mortality after extubation [M_aET] vs. survival after extubation [S_aET]). During comparisons between corresponding groups, numerical data were analyzed using an independent t-test, and categorical data were analyzed using the Chi-squared test and Fisher’s exact test. Candidate parameters were further entered into a multivariate logistic regression analysis for predictions. The statistical significance in the above analysis was denoted by two-tailed P-values of ≤ 0.05. All statistical analyses were performed using SPSS software (version 25.0, IBM, Armonk, NY, USA).

## Results

### Demographic characteristics of MC

The demographic characteristics of the 188 cases of MC are listed in Table 1. The age at MC onset was 55.59 ± 18.02 years. Sixty-two female and 33 male patients had ages of onset of <55 years, and 43 female and 50 male patients had ages of onset of ≥55 years (*p* = 0.009). During the couse of MC, five patients experienced acute myocardial infarction, and 17 cases suffered from acute kidney injury (AKI). Forty-five cases of MC occurred at the moment of MG diagnosis; therefore, they did not receive any medication for MG before hospitalization. Reviewing the medical treatment for MG before hospitalization in the other 143 cases, only symptomatic treatment with pyridostigmine was administered for 33 cases, and disease-modifying therapy (DMT) with or without prednisolone was administered for 110 cases. In the majority of MC events with DMT before hospitalization, prednisolone was used as monotherapy or in combination with other immunosuppressants (azathioprine or mycophenolate mofetil) for 103 cases. Plasma exchange or double-filter plasmapheresis (PE/DFPP) was performed for acute management for 109 cases. In this cohort, the mean length of hospitalization was 23.99 ± 15.64 days. Mortality during mechanical ventilation was identified in 27 patients, and 20 patients died within 2 weeks after intubation. Among 161 patients who survived at the time of the removal of the endotracheal tube, 12 cases needed mechanical ventilation for more than 14 days and 16 cases needed reintubation within three days. Fifteen patients died after extubation. The overall in-hospital mortality rate was 22% (42/188), and the reintubation rate was 10% (16/161).

**Table 1:** Demography of myasthenic crisis patients

	MC events (***n*** = 188)
Age at MC onset (years)	55.59 ± 18.02
Sex (Female / Male)	83 / 105
Underlying diseases	
Hypertension	52
Diabetes mellitus	36
Dyslipidemia	7
COPD	3
Liver cirrhosis	6
CRF	7
Acute comorbidity	
AMI	5
AKI	17
Medication for MG before admission	
None	45
Pyridostigmine only	33
DMT with or without pyridostigmine	110
Prednisolone monotherapy	68
Immunosuppressant monotherapy	7
Prednisolone + Immunosuppressant	35
PE / DFPP	109
Length of hospital stay (days)	23.99 ± 15.64
During mechanical ventilation	
Death (≦2 weeks after intubation / >2 weeks after intubation)	27 (20 / 7)
After removal of endotracheal tube	
Mechanical venntilation > 14 days	12
Reintubation within 3 days	16
Death (≦2 weeks after extubation / >2 weeks after extubation)	15 (9 / 6)
Survival at discharge	146

ѱ *Abbreviations*: *AKI* Acute kidney injury, *AMI* Acute myocardial infarction, *COPD* Chronic obstructive pulmonary disease, *CRF* Chronic renal failure, *DMT* Disease modified therapy, *MC* Myasthenic crisis, *PE / DFPP* Plasma exchange or double-filtration plasmapheresis

### Mortality during mechanical ventilation

Table 2 illustrates the comparison of the different outcomes during mechanical ventilation in MC patients. Twenty-six patients in the M_dMV group were significantly older at the onset of MG (p <0.001) than those in the S_dMV group. The MC patients with liver cirrhosis had a higher risk of mortality during mechanical ventilation than those without liver cirrhosis (p = 0.006). The latest hemogram before intubation showed that the Hb concentrations were significantly lower in patients in the M_dMV group than those in the S_dMV group (p <0.001), and moderate-to-severe anemia was identified in 61.5% of the patients in the M_dMV group and 21.6% of those in the S_dMV group (p <0.001). Patients in the M_dMV group had lower platelet counts than those in the S_dMV group (p <0.001), and thrombocytopenia was found in 50% of patients in the M_dMV group and 24.5% of those in the S_dMV group (p = 0.008). In addition, extreme leukocytosis was found in 19.2% of the patients in the M_dMV group and 6.5% of the patients in the S_dMV group (p = 0.032). There was no significant difference in the NLR of the latest hemogram before intubation between the M_dMV and S_dMV groups. During mechanical ventilation, the MC patients treated with PE/DFPP had a lower risk of mortality than those who did not receive treatment (p < 0.001).

**Table 2:** Comparison of the outcomes in MC patients during mechanical ventilation

	During mechanical ventilation				
	Mortality	Survival			
	M_dMV (*N* = 26)	S_dMV (*N* = 139)	Crude p	OR (95%CI)	Adjusted p
Age at MC onset (years)	66.73 ± 15	52.42 ± 18.14	<0.001*	1.039 (1.006-1.074)	0.022*
Sex					
Female / Male	12 / 14	83 / 56	0.199		
Underlying diseases [n (%)]					
Hypertension	3 (11.5)	36 (25.9)	0.136		
Diabetes mellitus	4 (15.4)	24 (17.3)	1.000		
Dyslipidemia	0 (0)	5 (3.6)	1.000		
COPD	0 (0)	3 (2.2)	1.000		
Liver cirrhosis	4 (15.4)	2 (1.4)	0.006*	6.19 (0.719-53.266)	0.097
CRF	1 (3.8)	5 (3.6)	1.000		
Medication for MG before admission [n (%)]					
Pyridostigmine only	5 (19.2)	24 (17.3)	0.809		
DMT with or without pyridostigmine	17 (65.4)	85 (61.2)	0.683		
Prednisolone monotherapy	11 (64.7)	50 (58.8)	0.080		
Immunosuppressant monotherapy	3 (17.6)	4 (4.7)			
Prednisolone + Immunosuppressant	3 (17.6)	31 (36.5)			
PE / DFPP [n (%)]	6 (23.1)	88 (63.3)	0.000*	0.236 (0.077-0.728)	0.012*
Hemogram in 3 days before intubation					
Hb (g/dL)	10.32 ± 2.7	12.66 ± 2.15	<0.001*		
Hb < 11 g/dL [n (%)]	16 (61.5)	30 (21.6)	<0.001*	5.851 (2.003-17.092)	0.001*
Platelet (x 10^9^/L)	140.88 ± 84.37	225.62 ± 106.56	<0.001*		
Platelet < 150 x 10^9^/L [n (%)]	13 (50)	34 (24.5)	0.008*	1.045 (0.349-3.126)	0.937
WBC (x 10^9^/L)	16.83 ± 12.3	13.9 ± 7.7	0.250		
WBC≧25 x 10^9^/L [n (%)]	5 (19.2)	9 (6.5)	0.032*	5.659 (1.28-25.023)	0.022*
Neutrophil (%)	79.88 ± 17.32	82.18 ± 12.31	0.415		
Lymphocyte (%)	13.65 ± 15.99	12.03 ± 9.25	0.620		
NLR	20.2 ± 22.59	14.66 ± 20.83	0.221		

ѱ *Abbreviations*: *COPD* Chronic obstructive pulmonary disease, *CRF* Chronic renal failure, *DMT* Disease modified therapy, *Hb* Hemoglobin, *MC* Myasthenic crisis, *M_dMV* Mortality during mechanical ventilation, *NLR* Neutrophil-lymphocyte ratio, *S_dMV* Survival during mechanical ventilation, *PE / DFPP* Plasma exchange or double-filtration plasmapheresis, *WBC* White blood count

The multivariate logistic regression analysis used to predict the mortality during mechanical ventilation showed that older age at MC onset was a risk factor for mortality (OR = 1.039, p = 0.022). Moderate-to-severe anemia (OR=5.851, p = 0.001) and extreme leukocytosis (OR=5.659, p = 0.022) within 3 days before intubation were also significant predictors of mortality, whereas acute management with PE/DFPP significantly decreased mortality (OR=0.236, p = 0.012). Liver cirrhosis and thrombocytopenia were not significantly associated with mortality within 3 days before intubation. The accuracy of this multivariate logistic regression model was 87.9% for predicting mortality during mechanical ventilation.

### Prediction of mortality after extubation

Table 3 illustrates the comparison of the different outcomes after extubation in patients with MC. The age of onset of MC in the M_aET group was significantly higher than that in the S_aET group (p = 0.012). The percentage of patients in the M_aET group who received only received pyridostigmine without disease-modifying therapy before MC (45.5%) was significantly higher than that in the S_aET group (14.2 %) (p = 0.009). The MC patients who suffered from acute kidney injury (AKI) during the MC had a higher risk of mortality after extubation than those without AKI (p = 0.018). The hemogram data before extubation showed that the Hb concentrations were significantly lower in the M_aET group than in the S_aET group (p = 0.006), and moderate-to-severe anemia was identified in 81.8% of patients in the M_aET group and 33% of patients in the S_aET group (p = 0.002). There was no significant difference in the NLR between the patients in the M_aET and S_aET groups.

**Table 3:** Comparison of the outcomes in MC patients after extubation

	After extubation				
	Mortality	Survival			
	M_aET (*N* = 11)	S_aET (*N* = 106)	Crude p	OR (95%CI)	Adjusted p
Age at MC onset (years)	66.27 ± 13.93	52.31 ± 17.51	0.012*	1.05 (0.997-1.104)	0.063
Sex					
Female / Male	6 / 5	63 / 43	0.754		
Underlying diseases [n (%)]					
Hypertension	2 (18.2)	29 (27.4)	0.725		
Diabetes mellitus	3 (27.3)	19 (17.9)	0.431		
Dyslipidemia	1 (9.1)	3 (2.8)	0.330		
COPD	0 (0)	3 (2.8)	1.000		
Liver cirrhosis	1 (9.1)	1 (0.9)	0.180		
CRF	1 (9.1)	2 (1.9)	0.258		
Acute comorbidity [n (%)]					
AMI	0 (0)	2 (1.9)	1.000		
AKI	3 (27.3)	4 (3.8)	0.018*	4.3 (0.65-28.457)	0.13
Medication for MG before admission [n (%)]					
Pyridostigmine only	5 (45.5)	15 (14.2)	0.009*	5.459 (1.17-25.472)	0.031*
DMT with or without pyridostigmine	4 (36.4)	63 (59.4)	0.201		
Prednisolone monotherapy	3 (75)	34 (54)	0.050		
Immunosuppressant monotherapy	1 (25)	2 (3.2)			
Prednisolone + Immunosuppressant	0 (0)	27 (42.9)			
PE / DFPP [n (%)]	5 (45.5)	65 (61.3)	0.307		
Hemogram in 3 days before extubation					
Hb (g/dL)	10.24 ± 1.14	11.81 ± 1.83	0.006*		
Hb < 11 g/dL [n (%)]	9 (81.8)	35 (33)	0.002*	8.452 (1.455-49.089)	0.017*
Platelet (x 10^9^/L)	202 ± 58.62	237.29 ± 112.39	0.308		
Platelet < 150 x 10^9^/L [n (%)]	2 (18.2)	22 (20.8)	1.000		
WBC (x 10^9^/L)	9.6 ± 2.93	13.75 ± 7.73	0.081		
WBC≧25 x 10^9^/L [n (%)]	0 (0)	8 (7.5)	1.000		
Neutrophil (%)	71.98 ± 25.56	81.86 ± 11.41	0.232		
Lymphocyte (%)	14.74 ± 13.53	12.12 ± 8.14	0.543		
NLR	11.36 ± 11.11	13.73 ± 23	0.736		
Reintubation within 3 days	3 (27.3)	10 (9.4)	0.105		
Mechanical ventilation more than 14 days	2 (18.2)	4 (3.8)	0.098		

ѱ *Abbreviations*: *AKI* Acute kidney injury, *AMI* Acute myocardial infarction, *COPD* Chronic obstructive pulmonary disease, *CRF* Chronic renal failure, *DMT* Disease modified therapy, *Hb* Hemoglobin, *MC* Myasthenic crisis, *M_aET* Mortality after extubation, *NLR* Neutrophil-lymphocyte ratio, *S_aET* Survival after extubation, *PE / DFPP* Plasma exchange or double-filtration plasmapheresis, *WBC* White blood count

The multivariate logistic regression analysis used to predict mortality after extubation showed that the presence of moderate-to-severe anemia before extubation (OR=8.452, p = 0.017) and non-treated with disease-modifying therapy before MC (OR=5.459, p = 0.031) were crucial predictors. Despite the statistical insignificance, older age at MC onset was associated with a higher risk of mortality after extubation for this model. Acute kidney injury had no significant effect on mortality after extubation. The multivariate logistic regression model showed a 93.2% predictive accuracy.

## Discussion

In our study, the mean age at the onset of MC was 55.59 years, which was similar to that for the American (58.8 years) and Chinese (52.5 years) populations [9, 32]. Although a bimodal distribution of the ages at the onset of MC has been reported in a previous study [10], there is no correspondence in this cohort. In our study, we found that MC patients demonstrated a female predominance before the age of 55 years and sexual equality after the age of 55 years, which suggests that the difference in the sexual distribution of MC patients is related to the age at onset. A similar presentation of differences in the sexual distribution has also been demonstrated in previous studies [10, 33]. MC can occur at any time during the course of MG [34], and it may also be the initial presentation of MG in one-fifth of patients [10]. Liu et al. [33] reported that the rate of first MC was 5.6%, but it was up to 24% (45/188) in our cohort. The difference may be contributed to the sources of patients enrolled. The MC patients in Liu’s study were enrolled from a single medical center in China, while those in our cohort were enrolled from multiple hospitals, including three medical centers and four regional hospitals in different areas of Taiwan.

The mortality rate of MC was 42% in the early 1960s [5], and it has decreased to 4–30% [9, 32, 35–37] in recent decades because of advances in mechanical ventilation and acute immunomodulatory therapies [7]. The in-hospital mortality rate of our cohort was 22% (42/188), which was lower than the 30% in an Indian report [36] but higher than those in China (15.9%) and Brazil (16.7%) [33, 35]. The discrepancy in mortality may be due to the selection of acute management for MC. In the case series of Indian and Brazil reports, >50% MC patients did not receive PE/DFPP or IVIg as acute management, while 67.3% MC patients received IVIg as acute management in the China report. However, the major acute management for MC in our cohort was PE/DFPP, which was afforded by the national health insurance in Taiwan. Because PE/DFPP have higher risk of leading to hemodynamic instability than IVIg, especially in critical patients, it could explain the difference in mortality rate in our cohort and China report.

Infection is a trigger event of MC [38], and it more frequently occurred in elderly MG patients [39]. Severe infection can lead to dehydration, AKI, and hemodynamic instability, which may further influence the choice of acute management of MC [40]. Among the 17 patients complicated with AKI in our cohort, absence of acute management due to severe infection was found in 13 patients, while age at MC onset > 60 years was identified in 11 patients. Based on these findings, we supposed that dehydration secondary to severe infection and elderly age was a causative factor of AKI in our MC patient. Extreme leukocytosis is associated with severe infection, cancer, and many acute medical illness, and it had also been proved as a robust predictor of mortality of patients in emergent department and intensive care unit [6, 41]. In our study, extremely leukocytosis was a crucial predictor of mortality during mechanical ventilation in MC patients.

Previous studies have shown that age is an independent risk factor for mortality in patients with MG, especially in those older than 50 years [8, 9, 42]. Liu et al. also reported that older age at onset for the first MC was predictive of a higher mortality risk in MC patients in the neurological intensive care unit [33]. In our study, older age at MC onset was an important predictor of mortality during mechanical ventilation and after extubation.

Anemia is an independent mortality predictor for different acute critical illnesses, including acute myocardial infarction [15, 16], infectious endocarditis [17], acute exacerbation of chronic obstructive pulmonary disease [18, 19], acute kidney injury [20], acute ischemic stroke [21], and community-acquired pneumonia [22]. Lv et al. also reported that the lowest Hb concentration was lower in the MC patients who died during hospitalization than in those who survived [32]. In this study, moderate-to-severe anemia (Hb < 11 g/dL) before intubation was an independent predictor of mortality during mechanical ventilation in MC patients, and moderate-to-severe anemia could also predict mortality after extubation.

Hemogram-derived indices are easily available markers of systemic inflammation and have been used as predictors of detrimental outcomes in several diseases [23–25]. Yang et al. also reported that NLR of >2.33 was an independent predictor of severe disease activity and MC in patients with MG [27]. In our study, an NLR of >2.33 was reported for 91% of cases before intubation, but there was no statistical difference in the NLR between the M_dMV and S_dMV groups. Similarly, an NLR of >2.33 was found in the latest hemogram before intubation in 93% of cases, but there was also no difference between the M_aET and S_aET groups.

Based on these findings, we concluded that both older age at MC onset and moderate-to-severe anemia could predict mortality during mechanical ventilation and after extubation. As a marker of systemic inflammation, NLR is commonly high in patients with MC, but it is not a predictor of in-hospital mortality. Respiratory failure is the major clinical manifestation of MC and can lead to hypoxia, while anemia can further exacerbated hypoxia. Liu et al. [43] reported that both hypoxia and systemic inflammation have adverse effects on MG, and several studies have also demonstrated that chronic and intermittent hypoxia can provoke systemic inflammation by activating hypoxia inducible factor-1 (HIF-1), and increasing the expression of proinflammatory factors, such as interleukin (IL)-2, IL-4, and interferon (IFN)-γ [44–49]. Excessive release of proinflammatory factors leading to uncontrolled systemic inflammation is evident in almost all patients with multiple organ dysfunction syndrome (MODS) and is an important prognostic factor for ICU survival [50]. Based on our findings and the evidence from previous studies, we hypothesized that anemia exacerbates hypoxia caused by respiratory failure in MC patients and hypoxia-related systemic inflammation, which further leads to MODS and eventually death.

Our study has some limitations. First, several important factors for MG classification (e.g., serology and thymic pathology) and dysfunction severity (e.g., quantitative MG score and MG composite scale) were not recorded in the electronic medical record database, and some of these factors may also be crucial mortality factors in MC patients. Second, hemogram data within 3 days before intubation and before extubation were not available for all patients, which may have resulted in selection bias. Third, no patients in our cohort received intravenous immunoglobulin; therefore, the impact of different acute management strategies on in-hospital mortality of MC patients is uncertain. Fourth, our study excluded patients younger than 18 years and those who used a ventilator for tracheostomy at home before admission, and it is necessary to conduct further research on the mortality of these patients. However, most previous studies on the outcome of MC involved retrospective analyses of patients from a single hospital, whereas our study is a retrospective study using a universal electronic medical record database of multiple hospitals, which contains data that are more similar to the real-world data of a country.

## Conclusion

This retrospective study focused on in-hospital mortality of MC patients and is based on data from a universal database from multiple hospitals in Taiwan, resulting in a higher ratio of first MC events and in-hospital mortality rates compared to previous studies. Both older age at onset and moderate-to-severe anemia are important predictors of in-hospital mortality in MC patients at different stages, and extreme leukocytosis is another crucial predictor of mortality during mechanical ventilation. The suggested mechanism of in-hospital mortality in MC patients is that anemia-induced hypoxia may enhance the release of proinflammatory cytokines, exacerbate systemic inflammation, and lead to MODS and, finally, death.

## Data Availability

The de-identified dataset generated and analyzed in this study is electronically stored and protected by specific password. The dataset is available from the corresponding author on reasonable request.
